# Pneumococcal Disease Outbreak at a State Prison, Alabama, USA, September 1–October 10, 2018^1^

**DOI:** 10.3201/eid2707.203678

**Published:** 2021-07

**Authors:** Guillermo V. Sanchez, Constance L. Bourne, Sherri L. Davidson, Mark Ellis, Leora R. Feldstein, Katherine Fay, Nicole E. Brown, Evelyn F. Geeter, Lytasha L. Foster, Charlotte Gilmore, Mary G. McIntyre, Burnestine Taylor, Srinivasan Velusamy, Sopio Chochua, Almea M. Matanock

**Affiliations:** Centers for Disease Control and Prevention, Atlanta, Georgia, USA (G.V. Sanchez, L.R. Feldstein, K. Fay, N.E. Brown, S. Velusamy, S. Chochua, A.M. Matanock);; Alabama Department of Public Health, Montgomery, Alabama, USA (G.V. Sanchez, C.L. Bourne, S.L. Davidson, M. Ellis, E.F. Geeter, L.L. Foster, C. Gilmore, M.G. McIntyre, B. Taylor)

**Keywords:** pneumococcus, correctional facility, outbreak, PPSV23, azithromycin, meningitis/encephalitis, *Streptococcus pneumoniae*, Alabama, USA, pneumococcal disease

## Abstract

A pneumococcal disease outbreak caused by *Streptococcus pneumoniae* serotype 12F occurred in a state prison in Alabama, USA. Among 1,276 inmates, 40 cases were identified (3 confirmed, 2 probable, 35 suspected). Close living quarters, substance use, and underlying conditions likely contributed to disease risk. Prophylaxis for close contacts included azithromycin and 23-valent pneumococcal polysaccharide vaccine.

*Streptococcus pneumoniae* (pneumococcus) causes a spectrum of disease ranging from mild respiratory infections to severe disease, including meningitis, sepsis, and pneumonia (*1*). Invasive pneumococcal disease (IPD) occurs when pneumococcus invades normally sterile sites. Pneumococcus is transmitted person-to-person primarily through respiratory droplets and is a leading cause of vaccine-preventable illness and death (*2*). Pneumococcal colonization is a precursor to disease but does not always result in disease (*3*). Pneumococcal conjugate vaccine (PCV) is highly effective in preventing pneumonia in adults (*4*), and pneumococcal disease incidence has declined since the introduction of PCV (*5*). IPD outbreaks are rare but can occur in settings with close person-to-person contact, such as homeless shelters (*6*) and healthcare facilities, in which underlying conditions can increase disease risk (*7*).

On September 19, 2018, the Alabama Department of Public Health (Montgomery, AL) was notified of an IPD case after identification of *S. pneumoniae* in a blood culture from an ill patient incarcerated at a state prison. On September 24, a second case of IPD was reported in another inmate who received a diagnosis of meningitis and sepsis and died that morning. We investigated this outbreak to determine its extent, identify cases among staff and inmates, and recommend prophylactic measures to reduce spread.

## The Study

At the time of the outbreak, facility A, a medium-security state prison, housed 1,276 male inmates across 6 dormitories (original capacity 650 inmates; 2018 reported capacity of 1,650 inmates) (*8*,*9*). Each dormitory contained multiple large rooms with 4–6 rows of beds for 190–255 inmates. Group activities allowed mixing of inmates from different dorms until the outbreak was recognized; activities were suspended around September 26. A clinic within facility A with a 52-member staff, including 2 nurse practitioners and a physician, provided services to inmates through self or employee referral.

A suspected case was defined as respiratory or meningeal symptoms consistent with pneumococcal disease in an incarcerated person or a person in prolonged or close contact with anyone incarcerated at facility A during September 1–October 10, 2018 (Appendix). Probable cases were defined as suspected cases with radiographic-confirmed pneumonia, clinical sepsis, or cerebrospinal fluid analysis suggestive of bacterial meningitis with unknown etiology. Confirmed cases were defined as suspected cases with *S. pneumoniae* isolation or positive urinary antigen test.

We conducted retrospective case finding among inmates who were seen in the clinic during September 1–September 29 and prospective surveillance during September 30–October 10. Cases in which inmates reported respiratory illness, altered mental status, headache, or fever and those without a listed chief complaint were flagged for medical chart review. Inmates whose medical records indicated signs or symptoms of pneumococcal disease were interviewed by using a standardized questionnaire to identify clinical characteristics, risk factors, and epidemiologic links with confirmed cases. For prospective surveillance, we screened all inmates who experienced respiratory symptoms for pneumococcus and influenza by using nasopharyngeal swabs. We tested influenza-negative swab specimens for other respiratory pathogens by using the BioFire FilmArray Respiratory Panel (bioMérieux, https://www.biomerieux.com). We performed pneumococcal serotyping on nasopharyngeal swab specimens from which *S. pneumoniae* was isolated. We calculated attack rates by dividing the number of identified cases by the at-risk population (i.e., all dormitory residents). Specimen culture and antibiotic susceptibility testing was performed at laboratories in the hospitals in which patients received care and confirmed at the Centers for Disease Control and Prevention. Sterile body fluids and nasopharyngeal swab specimens were sent to Centers for Disease Control and Prevention for species detection, confirmation, and serotyping by using real-time reverse transcription PCR. Bacterial serotyping and whole-genome sequencing (WGS) were performed on pneumococcal isolates collected from confirmed cases. We analyzed single-nucleotide polymorphisms identified through WGS to verify temporal relatedness of the isolates.

Through retrospective case finding, 96 medical chart reviews and 52 inmate interviews identified 40 cases (3 confirmed, 2 probable, and 35 suspected; Figure) for attack rates of 3% (40/1,276) within facility A and 5% (14/255) within dormitory X. All confirmed cases occurred in inmates living in dormitory X. Suspected cases were identified among inmates in all 6 dormitories. No pattern of temporal spread was observed among dormitories. Of suspected cases, 26% (9/35) reported lower respiratory symptoms (chest pain, shortness of breath, or fever with cough) (Table 1). Underlying conditions for which 23-valent pneumococcal polysaccharide vaccine (PPSV23) is routinely recommended (excluding smoking tobacco) (*10*) were reported in 2/5 (40%) confirmed or probable cases and 6/35 (17%) suspected cases. In 3/35 (9%) suspected cases, patients were immunocompromised. Of the 28/40 (70%) patients who reported smoking cigarettes, half (14/28, 50%) reported sharing cigarettes.

**Figure:**
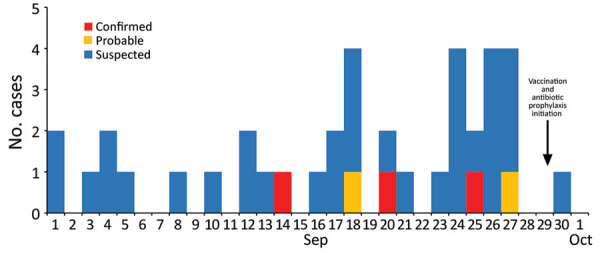
Epidemic curve of identified cases among inmates in study of pneumococcal disease outbreak at a state prison, Alabama, USA, September 1–October 10, 2018. Date of first symptom onset is shown. Healthcare unit visit date was used when symptom onset date was not known. Two suspected cases without a clear onset date were excluded from this graph.

**Table 1:** Patient demographics, signs, symptoms, and *Streptococcus pneumoniae* risk factors by case classification in study of pneumococcal disease outbreak at a state prison, Alabama, USA, September 1–October 10, 2018*

Demographics	Confirmed cases, n = 3		Probable cases, n = 2		Suspected cases, n = 35	
Median age, y (range)	46 (44–61)		42 (26–58)		39 (23–64)	
Race						
Black	3 (100)		0		14 (40)	
White	0		2 (100)		13 (37)	
Unknown	0		0		8 (23)	
Signs or symptoms						
Fever	2 (67)		1 (50)		15 (43)	
Cough	2 (67)		1 (50)		19 (54)	
Shortness of breath	1 (33)		2 (100)		4 (11)	
Chest pain	1 (33)		1 (50)		6 (17)	
Headache	1 (33)		0		11 (31)	
Neck stiffness	2 (67)		0		1 (3)	
Altered mental status	3 (100)		1 (50)		0	
Congestion	0		0		6 (17)	
Clinical features and risk factors						
Immunocompromising condition†	0		0		3 (9)	
Chronic medical condition‡	1 (33)		1 (50)		6 (17)	
Substance use: cigarettes, alcohol, or illicit drugs	2 (67)		0		27 (77)	
Assigned housing						
Dormitory X	3 (100)		0		11 (31)	
Dormitory Y	0		1 (50)		8 (23)	
Other dormitories: U, V, W, Z	0		0		16 (46)	
Healthcare unit	0		1 (50)		0	

*Values are no. (%) except as indicated. Table includes composite statistics from both inmate interviews and medical chart abstractions. All identified cases occurred in inmates; no cases were identified among staff members. †Includes immune suppression, chronic renal failure, HIV, solid organ transplant, asplenia, sickle cell disease and other hemoglobinopathies, and malignancy excluding skin cancer (Centers for Disease Control and Prevention Adult Vaccine Schedule, https://www.cdc.gov/vaccines/acip). ‡Includes chronic heart, liver, kidney, lung diseases, and diabetes mellitus; excludes documented substance abuse, Hepatitis C infection, and chronic osteomyelitis (Centers for Disease Control and Prevention Adult Vaccine Schedule, https://www.cdc.gov/vaccines/acip).

Blood (n = 5) or cerebrospinal fluid (n = 4) specimens were collected from 5 patients. Three *S. pneumoniae* isolates were identified from 3 patients (2 from cerebrospinal fluid, 1 from blood); all 3 were serotype 12F. WGS demonstrated a 2–5 single-nucleotide polymorphism difference among isolates, indicating all confirmed cases were closely related. Antimicrobial susceptibility testing confirmed isolate susceptibility.

During prospective surveillance, we collected nasopharyngeal swab specimens from 2 inmates; *S. infantis* serotype 13 was detected in 1 specimen and adenovirus only in the other. No additional cases were identified. Prophylaxis with PPSV23, which protects against serotype 12F, and 2 doses of azithromycin were offered to inmates and close contacts living in or assigned to dormitory X, clinic staff, and prison employees (Table 2).

**Table 2:** Administration of pneumococcal polysaccharide vaccine and antibiotic prophylaxis to reduce *Streptococcus pneumoniae* transmission in prison inmates during a pneumococcal disease outbreak, Alabama, USA, September 29–October 10, 2018*

Prophylaxis type	Status	No. (%)		
		Inmates, n = 264†	Medical staff, n = 52	Prison employees, n = 72
PPSV23 vaccine	Received	206 (78)	32 (62)	62 (86)
	Declined	58 (22)	18 (35)	8 (11)
	Absent or unknown	0	2 (4)	2 (3)
Dose 1: azithromycin 500 mg‡	Received	232 (88)	11 (21)	70 (97)
	Declined	15 (6)	6 (12)	0
	Absent or unknown	17 (6)	35 (67)	2 (3)
Dose 2: azithromycin 1000 mg	Received	246 (93)	35 (67)	46(64)
	Declined	18 (7)	16 (31)	4 (6)
	Absent or unknown	0	1 (2)	22 (31)

*Because of rounding, all cells might not sum to 100%. PPSV23, 23-valent pneumococcal polysaccharide vaccine. †Inmates assigned to dormitory X (n = 255) or living in dormitory X without an assignment (n = 9) were offered prophylaxis. Not all inmates lived or slept in the dorm to which they were assigned. ‡Initial antibiotic prophylaxis dosing was chosen by the Alabama Department of Corrections as 500 mg offered on September 29 and a planned second dose of 500 mg 1 week later. This dose would have provided less antibiotic coverage than previous outbreak regimens. Based on Alabama Department of Health recommendations, a higher, single dose of azithromycin (1,000 mg) was offered during October 3–October 10.

## Conclusions

This investigation highlights the outbreak potential of *S. pneumoniae* and demonstrates that correctional facilities remain at risk for pneumococcal outbreaks after PCV introduction in the United States. The last documented pneumococcal disease outbreak at a US correctional facility also involved serotype 12F and occurred in 1989 in a crowded Texas jail, in which 46 inmates experienced pneumonia, meningitis, or sepsis over a 4-week period (*11*). Unlike jails, which have an average detention length of <2 months, prisons have lower rates of inmate turnover because they are designed for long-term incarceration; the average detention among US state prisoners in 2016 was 2.6 years (*12*). However, jails and prisons are similar in that they share risk factors for outbreaks, such as potential overcrowding and increased medical and behavioral risk factors for communicable diseases (*13*). Decreases in pneumococcal disease have been observed during outbreaks after administering prophylactic antibiotics and PPSV23 to high-risk persons (*6*,*7*,*11*,*14*). Risk for pneumococcal disease outbreaks in prisons can be minimized by offering inmates vaccinations per Advisory Committee on Immunization Practices recommendations, which recommend PPSV23 for persons with chronic heart, liver, or lung disease (*15*). Risk can be further reduced by minimizing inmate crowding, eliminating indoor smoking, and ensuring adequate ventilation (*11*).

One limitation of this study was that risk factor information was collected by self-reporting and might be underestimated. Disease etiology was not confirmed for most cases because few patients had confirmatory laboratory testing for respiratory pathogens. Dormitories were not assessed for space, capacity, or ventilation.

Pneumococcal colonization among inmates could not be widely assessed. We observed decreases in pneumococcal disease after prophylaxis administration, but we cannot determine the direct impact of prophylaxis since *S. pneumoniae* serotype 12F carriage was not measured. Pneumococcal carriage studies among incarcerated populations could further our understanding of pneumococcal disease in correctional facilities.

In our outbreak investigation of pneumococcal disease in a state prison, we observed decreases in disease after prophylaxis with PPSV23 and azithromycin. Increased pneumococcal disease risk might have resulted from close living quarters, substance use, and underlying conditions. Improved pneumococcal disease surveillance and proactive vaccination of at-risk inmates in accordance with Advisory Committee on Immunization Practices recommendations might mitigate risk for and scale of future outbreaks.

Appendix Additional information about pneumococcal disease outbreak at state prison, Alabama, USA, September 1–October 10, 2018
