# Supplementary material for: Pneumococcal Disease Outbreak at a State Prison, Alabama, USA, September 1–October 10, 2018
Source: Emerg Infect Dis. 2021 Jul;27(7):1949–52. doi: 10.3201/eid2707.203678 (PMC8237874; doi:10.3201/eid2707.203678)
Supplement: Appendix — Additional information about pneumococcal disease outbreak at state prison, Alabama, USA, September 1–October 10, 2018 [file 20-3678-Techapp-s1.pdf]

Article DOI: https://doi.org/10.3201/eid2707.203678

Pneumococcal Disease Outbreak at State Prison, Alabama, USA, September 1–October 10, 2018

Appendix

Case Definitions for Pneumococcal Disease

Case Definitions: Identification of the following signs and symptoms occurring on or after September 1, 2018 in a person who has been incarcerated or who has had prolonged or close contact with a person incarcerated in Facility A:

1. Suspected pneumococcal case:
    - a. Suspected pneumonia
        - i. Pneumonia diagnosed by clinician but no radiographic studies obtained or radiographs were negative
    - b. Symptomatic respiratory infection
        - i. $\geq$2 below, without meeting any of the above case definitions:
            1. Fever $\geq$38°C
            2. Cough
            3. Chest pain
            4. Difficulty breathing
            5. Sore throat
            6. Ear pain
    - c. Symptomatic bacterial meningitis
        - i. $\geq$2 below, without meeting any of the above case definitions:

            1. Fever ≥38°C
            2. Neck stiffness
            3. Altered mental status
            4. Other meningeal signs

2. Probable pneumococcal case:
    a. Radiographically confirmed pneumonia (either chest radiography or computed tomography scan) or clinical sepsis without laboratory evidence of *Streptococcus pneumoniae* (i.e., in the absence of the laboratory tests listed above)
    b. Laboratory evidence of bacterial meningitis
        i. Cerebrospinal fluid without *S. pneumoniae* identified, but with any of the following features:
            1. Turbid appearance
            2. Leukocytosis (>100 cells mm^3)
            3. Leukocytosis (10–100 cells mm^3) AND either an elevated protein (>100mg/dL) or decreased glucose (<40mg/dL)

3. Confirmed pneumococcal case:
    a. Invasive pneumococcal disease
        i. *S. pneumoniae* isolated from a normally sterile site (e.g., blood, cerebrospinal fluid, pleural fluid)
    b. Noninvasive pneumococcal disease
        i. Clinical illness compatible with *S. pneumoniae* (radiographically confirmed or clinically diagnosed pneumonia or otitis media) and isolation of *S. pneumoniae* from a nonsterile site (e.g., endotracheal specimen, sputum, otorrhea, nasal swab) or positive pneumococcal urinary antigen test in the absence of 23-valent pneumococcal polysaccharide vaccine receipt in the last 5 days
